# Machine learning–based prediction of mortality and hospitalization in diabetic patients with heart failure with preserved ejection fraction: the GUARDIAN-P risk score

**DOI:** 10.1093/ehjdh/ztag083

**Published:** 2026-06-10

**Authors:** Zheng-Wei Chen, Jen-Fang Cheng, Chen-Yu Huang, Tin-Tse Lin, Ting-Chuan Wang, Yen-Yun Yang, Shu-Lin Chuang, Chia-Ti Tsai, Lian-Yu Lin, Chung-Lieh Hung, Cho-Kai Wu

**Affiliations:** Division of Cardiology, Department of Internal Medicine, National Taiwan University College of Medicine and Hospital, No. 7, Chung-Shan South Road, Taipei City 100, Taiwan; Division of Cardiology, Department of Internal Medicine, National Taiwan University Hospital, Yun-Lin Branch, No. 579, Section 2, Yunlin Road, Douliu City, Yunlin County 640, Taiwan; Graduate Institute of Clinical Medicine, College of Medicine, National Taiwan University, No. 7, Chung-Shan South Road, Taipei City 100, Taiwan; Cardiovascular Center, National Taiwan University Hospital, No. 7, Chung-Shan South Road, Taipei City 100, Taiwan; Division of Cardiology, Department of Internal Medicine, National Taiwan University College of Medicine and Hospital, No. 7, Chung-Shan South Road, Taipei City 100, Taiwan; Cardiovascular Center, National Taiwan University Hospital, No. 7, Chung-Shan South Road, Taipei City 100, Taiwan; Division of Hospitalist, Department of Internal Medicine, National Taiwan University College of Medicine and Hospital, No. 7, Chung-Shan South Road, Taipei City 100, Taiwan; Graduate Institute of Clinical Medicine, College of Medicine, National Taiwan University, No. 7, Chung-Shan South Road, Taipei City 100, Taiwan; Cardiovascular Center, Cathay General Hospital, No. 280, Section 4, Renai Road, Taipei City 106, Taiwan; School of Medicine, College of Life Science and Medicine, National Tsing Hua University, No. 101, Section 2, Kuang Fu Road, Hsinchu City 300, Taiwan; Division of Cardiology, Department of Internal Medicine, National Taiwan University College of Medicine and Hospital, No. 7, Chung-Shan South Road, Taipei City 100, Taiwan; Cardiovascular Center, National Taiwan University Hospital, No. 7, Chung-Shan South Road, Taipei City 100, Taiwan; Department of Medical Research, National Taiwan University Hospital, No. 7, Chung-Shan South Road, Taipei City 100, Taiwan; Department of Medical Research, National Taiwan University Hospital, No. 7, Chung-Shan South Road, Taipei City 100, Taiwan; Department of Medical Research, National Taiwan University Hospital, No. 7, Chung-Shan South Road, Taipei City 100, Taiwan; Division of Cardiology, Department of Internal Medicine, National Taiwan University College of Medicine and Hospital, No. 7, Chung-Shan South Road, Taipei City 100, Taiwan; Cardiovascular Center, National Taiwan University Hospital, No. 7, Chung-Shan South Road, Taipei City 100, Taiwan; Division of Cardiology, Department of Internal Medicine, National Taiwan University College of Medicine and Hospital, No. 7, Chung-Shan South Road, Taipei City 100, Taiwan; Cardiovascular Center, National Taiwan University Hospital, No. 7, Chung-Shan South Road, Taipei City 100, Taiwan; Division of Cardiology, Departments of Internal Medicine, Mackay Memorial Hospital, No. 92, Section 2, Chung-Shan North Road, Taipei City 104, Taiwan; Department of Medicine, Mackay Medical College, No. 46, Section 3, Zhongzheng Road, New Taipei City 252, Taiwan; Nursing and Management, Mackay Junior College of Medicine, No. 92, Shengjing Road, Taipei City 112, Taiwan; Institute of Biomedical Sciences, Mackay Medical College, No. 46, Section 3, Zhongzheng Road, New Taipei City 252, Taiwan; Division of Cardiology, Department of Internal Medicine, National Taiwan University College of Medicine and Hospital, No. 7, Chung-Shan South Road, Taipei City 100, Taiwan; Cardiovascular Center, National Taiwan University Hospital, No. 7, Chung-Shan South Road, Taipei City 100, Taiwan

**Keywords:** Heart failure with preserved ejection fraction (HFpEF), Diabetes mellitus, Machine learning, Risk prediction

## Abstract

**Aims:**

Diabetes mellitus (DM) is a major contributor to adverse outcomes in patients with heart failure with preserved ejection fraction (HFpEF). We aim to develop and externally validate a machine learning-based model using a random survival forest (RSF) approach for predicting the composite outcome of hospitalization for heart failure (HHF) and cardiovascular (CV) death in patients with DM and HFpEF.

**Methods and results:**

This retrospective cohort study included 1450 adult patients with coexisting DM and HFpEF identified from the National Taiwan University Hospital-Integrated Medical Database. An initial RSF model was trained using 27 clinical variables, and the top 9 predictors were selected to construct a parsimonious final model. Predictive performance was evaluated using the area under the receiver operating characteristic curve (AUC), and external validation was conducted in an independent cohort (*n* = 729) from MacKay Memorial Hospital. Over a mean follow-up of 3.6 ± 3.0 years, 327 patients (22.6%) experienced the composite outcome. The final RSF model achieved an AUC of 88.2% in the training cohort and 79.8% in the validation cohort. The nine selected predictors were age, N-terminal pro-brain natriuretic peptide, serum albumin, fasting glucose, estimated glomerular filtration rate, uric acid, left atrial diameter, peripheral artery disease, and left ventricular ejection fraction. Risk increased progressively with the number of risk factors present.

**Conclusion:**

The RSF-based model incorporating nine routinely available variables accurately predicts HHF and CV death in patients with DM and HFpEF. This tool may support personalized risk assessment and guide clinical decision-making.

## Graphical Abstract

**Graphical Abstract:**
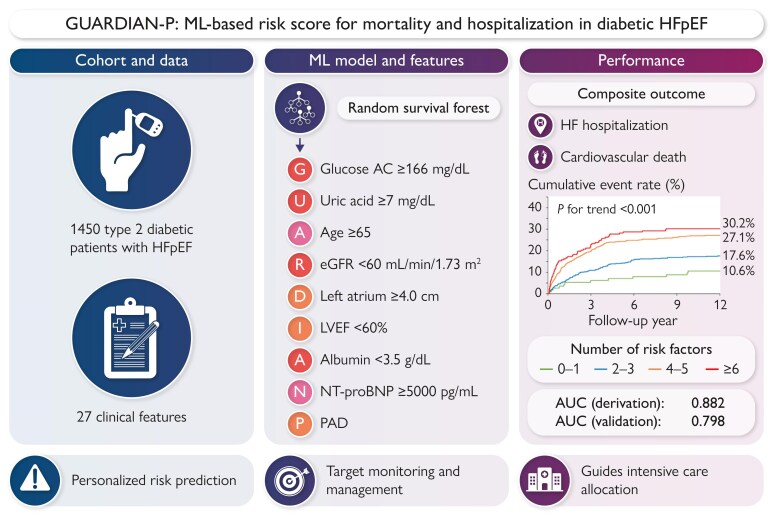

## Introduction

Heart failure (HF) represents a major global health burden, with rising prevalence, morbidity, and mortality.^1^ Heart failure with preserved ejection fraction (HFpEF) comprises approximately half of all HF cases and is characterized by preserved systolic function but impaired diastolic relaxation and compliance.^2^ Despite advances in understanding its pathophysiology, HFpEF remains a challenging clinical entity with limited therapeutic options and persistently poor outcomes.

HFpEF is a highly heterogeneous syndrome with multiple cardiometabolic phenotypes. In Asian populations, HFpEF often differs from Western cohorts, with patients generally being younger, less obese, and more frequently affected by metabolic comorbidities such as diabetes mellitus (DM) and chronic kidney disease (CKD).^3^ Particularly, a ‘lean diabetes’ phenotype has been increasingly recognized in Asian HFpEF populations, suggesting that metabolic dysfunction rather than obesity may play a dominant role in disease progression.^4^

DM is a highly prevalent comorbidity in patients with HFpEF, present in up to 45% of cases,^5^ and is independently associated with a 30–50% increased risk of mortality in this population.^6^ Additionally, the prevalence of DM among patients with HFpEF ranges from 22% to 40%.^7^ The global prevalence of DM continues to rise, driven by aging populations, lifestyle factors, and increasing rates of obesity, which will likely further increase the burden of HFpEF. The pathophysiological interplay between DM and HFpEF is complex and multifactorial, involving genetic, metabolic, and cellular mechanisms. DM contributes to impaired myocardial relaxation through mechanisms such as mitochondrial dysfunction, oxidative stress, and microvascular dysfunction, leading to myocardial fibrosis, hypertrophy, and diastolic dysfunction.^8^ Furthermore, neurohormonal activation, heightened systemic inflammation, and metabolic derangements exacerbate myocardial stiffness and increase susceptibility to adverse outcomes.^9^

Traditional prognostic models for HFpEF often neglect the nuanced contribution of DM and rely on linear associations of clinical and laboratory variables, potentially overlooking the heterogeneity of this patient population. Machine learning (ML) techniques, particularly random survival forests (RSF), offer an opportunity to capture the complex, nonlinear interactions between demographic, clinical, echocardiographic, and laboratory data. RSF models can handle high-dimensional data and right-censored survival outcomes, making them especially suitable for predicting mortality and hospitalization in patients with HFpEF and DM.^10^

This study aims to develop and validate an ML-based RSF model to predict mortality and hospitalization in diabetic patients with HFpEF. By leveraging comprehensive clinical data, including echocardiographic parameters and comorbidity profiles, the proposed model seeks to enhance risk stratification and inform personalized management strategies for this high-risk population. Our work integrates contemporary ML approaches with insights into the unique pathophysiology of diabetes-associated HFpEF, addressing an unmet need for precision risk prediction in this growing patient cohort.

## Methods

### Data sources and study design (summary)

This retrospective cohort study utilized electronic health records from the NTUH-Integrated Medical Database (NTUH-iMD), incorporating data from NTUH and its regional branches. Patients with HFpEF and type 2 DM were identified based on diagnostic codes, echocardiographic findings, medication records, and laboratory criteria. Comprehensive information on cohort selection, data extraction, and definitions of comorbidities and variables is provided in the Supplementary material (including Supplementary material online, *Figure S1* for the patient selection flowchart). The study was approved by the NTUH Research Ethics Committee (202306139RINC).

### Identification of the HFpEF with diabetes mellitus cohort

Patients with HFpEF and DM were identified based on electronic health records from the NTUH-iMD database. Eligible patients included adults (aged ≥20 years) who had a confirmed diagnosis of HF documented in inpatient, outpatient, or emergency department records at NTUH or its Hsin-Chu and Yun-Lin branches between 1 July 2006 and 31 May 2022. Patients were required to have undergone echocardiography within one year prior to diagnosis. HFpEF diagnosis was defined using a combination of clinician-assigned diagnostic codes and supporting clinical evidence from the electronic health record. To enhance diagnostic specificity, patients were required to have a documented HF diagnosis, echocardiographic confirmation of preserved left ventricular ejection fraction (LVEF ≥50%), and prescription of diuretics within ±1 month of the diagnosis.^11^ The diagnosis of DM was based on relevant diagnostic codes and further validated by either an HbA1c level ≥ 6.5% or the use of antidiabetic medications. The patient selection flowchart is presented in Supplementary material online, *Figure S1*. The index date was defined as either the date when HF was first diagnosed or the date of initial diuretic prescription, whichever occurred earlier. Demographic characteristics were obtained from the physical examination records closest to the index date. Comorbidities were defined based on diagnostic codes recorded within six months prior to the index date. A history of coronary artery bypass graft (CABG) was identified using Taiwan National Health Insurance (NHI) reimbursement codes. CKD was defined by either a corresponding diagnostic code or an estimated glomerular filtration rate (eGFR) below 60 mL/min/1.73m^2^ to maximize case ascertainment. Echocardiographic parameters were extracted from studies performed within one year before the index date, with the record closest to the index date selected if multiple were available. Laboratory test results and medication records were retrieved from data collected within one year prior to and six months following the index date, again selecting the value nearest to the index date in cases of multiple entries. All study variables were extracted electronically from structured fields within the NTUH-iMD. The inclusion criteria for the validation cohort were identical to those used for the training cohort, and detailed information regarding the validation cohort is provided in the Supplementary material.

### Outcome measurement and follow-up

The primary outcome measured was a composite of hospitalization for heart failure (HHF) and cardiovascular (CV) death. HHF was defined as an unscheduled admission during which intravenous diuretics, nitrites, or inotropic agents were administered,^12^ or the dose of oral diuretics was increased compared to the outpatient dosage prior to admission (including cases where diuretics were newly initiated during hospitalization). Using encrypted patient identifiers, the database was linked to the Taiwan National Death Registry, allowing precise identification of mortality outcomes. Information on both the date and cause of death was retrieved through this linkage. CV death was defined as death resulting from acute myocardial infarction, sudden cardiac death, HF, stroke, CV procedures, CV haemorrhage, or other CV-related causes.^13^ The primary analysis was conducted as a time-to-first event analysis. Patients were followed from the index date until the first occurrence of the composite outcome (HHF or CV death) or the end of the study period (30 November 2022), whichever occurred first. Only the first event per patient was included in the RSF modelling. The individual components of the composite endpoint and all-cause death were summarized descriptively to illustrate event composition during follow-up (see Supplementary material online, *Table S1*). All-cause mortality and all-cause hospitalization were not included in the predictive model to avoid heterogeneity driven by non-CV mechanisms and conditions not directly related to HF. To minimize the influence of acute clinical deterioration and ensure that outcomes reflected risk associated with baseline characteristics, patients with a follow-up duration of less than 30 days and events occurring within 30 days of the index date were excluded from the analysis.

### RSF analysis

Twenty-seven candidate variables (*Table 1*) were included in the initial RSF model, excluding parameters with high missingness (e.g. E/A ratio, E/e’), and selecting clinically representative variables. The RSF algorithm generated 500 survival trees using recursive splitting and ensemble hazard estimation. Variable importance (VIMP) guided stepwise model reduction, retaining predictors with VIMP ≥1% to balance performance and simplicity.^14^ The final 9-variable model achieved an AUC of 88.2% in the training cohort (*Table 2*). Detailed predictive behaviour and justification for variable selection are provided in the Supplementary material.

**Table 1:** Baseline characteristics in patients with diabetes and HFpEF who suffered from heart failure outcome (the composite of heart failure hospitalization and cardiovascular death) and who did not in the training sample (National Taiwan University Hospital)

Variable	Available number	Total (*n* = 1450)	Event (*n* = 327)	Event-free (*n* = 1123)	*P* value
Demographics					
Male sex	1450	721 (49.7)	156 (47.7)	565 (50.3)	0.407
Age, year	1450	73.5 ± 11.8	74.9 ± 11.3	73.1 ± 11.9	0.013
Body mass index, kg/m^2^	1408	25.7 ± 4.9	25.5 ± 4.6	25.7 ± 4.9	0.644
Systolic blood pressure, mmHg	1408	135.8 ± 22.0	135.1 ± 21.9	136.0 ± 22.0	0.520
Diastolic blood pressure, mmHg	1408	72.0 ± 13.5	71.8 ± 13.4	72.0 ± 13.5	0.852
Comorbidity					
Hypertension	1450	847 (58.4)	208 (63.6)	639 (56.9)	0.030
Hyperlipidaemia	1450	504 (34.8)	123 (37.6)	381 (33.9)	0.218
Coronary artery disease	1450	546 (37.7)	140 (42.8)	406 (36.2)	0.029
Peripheral artery disease	1450	155 (10.7)	46 (14.1)	109 (9.7)	0.025
Myocardial infarction	1450	110 (7.6)	31 (9.5)	79 (7.0)	0.142
Previous coronary artery bypass graft	1450	62 (4.3)	10 (3.1)	52 (4.6)	0.216
Chronic kidney disease	1450	28 (1.9)	218 (66.7)	664 (59.1)	0.014
Atrial fibrillation	1450	256 (17.7)	71 (21.7)	185 (16.5)	0.029
Chronic obstructive pulmonary disease	1450	68 (4.7)	8 (2.4)	60 (5.3)	0.029
Cerebrovascular disease (stroke or TIA)	1450	279 (19.2)	72 (22.0)	207 (18.4)	0.148
Laboratory data					
Fasting glucose, mg/dL	1240	135.6 ± 51.4	138.9 ± 55.1	134.6 ± 50.2	0.215
HbA1c, %	1450	7.0 ± 1.3	7.1 ± 1.4	6.9 ± 1.2	0.129
Creatinine, mg/dL	1445	2.0 ± 1.9	2.1 ± 1.8	2.0 ± 1.9	0.676
eGFR, mL/min/1.73m^2^	1445	53.5 ± 34.1	50.3 ± 33.8	54.4 ± 34.2	0.055
Albumin, g/dL	1204	3.6 ± 0.6	3.6 ± 0.6	3.6 ± 0.6	0.954
ALT, U/L	1413	17.0 [11.0, 26.0]	16.0 [11.0, 26.0]	17.0 [11.0, 27.0]	0.408
AST, U/L	1199	23.0 [17.0, 33.0]	23.0 [18.0, 31.0]	22.0 [17.0, 33.0]	0.809
AST/ALT ratio	1187	1.36 [1.00, 1.92]	1.44 [1.00, 1.94]	1.36 [0.96, 1.90]	0.311
Total cholesterol, mg/dL	1214	161.0 ± 44.1	160.4 ± 41.0	161.2 ± 45.0	0.777
High-density lipoprotein, mg/dL	1052	41.5 ± 13.1	40.7 ± 13.2	41.7 ± 13.0	0.308
Low-density lipoprotein, mg/dL	1097	89.3 ± 31.5	87.4 ± 27.0	89.8 ± 32.7	0.288
Triglyceride, mg/dL	1282	141.4 ± 84.9	135.6 ± 82.1	143.1 ± 85.6	0.183
Uric acid, mg/dL	1136	7.0 ± 2.5	7.5 ± 2.4	6.8 ± 2.5	0.000
hs-CRP, mg/dL	1018	1.6 [0.6, 4.3]	1.5 [0.6, 3.6]	1.7 [0.6, 4.5]	0.371
NT-proBNP, pg/mL	1020	2430 [765, 6812]	3266 [1156, 9210]	2279 [689, 6040]	0.002
Echocardiography laboratory data					
LA size, cm	1156	4.1 ± 0.8	4.3 ± 0.8	4.1 ± 0.8	0.001
Left ventricular end-diastolic diameter, cm	1204	4.8 ± 0.7	4.7 ± 0.6	4.8 ± 0.7	0.189
Left ventricular end-systolic diameter, cm	1204	3.0 ± 0.6	3.0 ± 0.5	3.0 ± 0.6	0.979
Left ventricular mass index, g/m^2^	1199	127.2 ± 35.4	130.3 ± 37.7	126.4 ± 34.7	0.111
Left ventricular ejection fraction, %	1450	65.5 ± 8.6	64.7 ± 9.0	65.7 ± 8.5	0.065
E/A ratio	914	1.0 ± 0.6	1.1 ± 0.5	1.0 ± 0.6	0.264
E/e’(medial), cm/s	328	18.9 ± 9.2	16.8 ± 6.5	19.2 ± 9.5	0.107
E/e’(lateral), cm/s	398	14.8 ± 8.2	13.7 ± 5.6	15.0 ± 8.6	0.221
Tricuspid regurgitation peak gradient, mmHg	1118	32.7 ± 13.8	34.2 ± 13.4	32.3 ± 13.9	0.0502
Left ventricular hypertrophy	1415	1049 (74.1)	244 (76.3)	805 (73.5)	0.326
Follow-up year	1450	3.6 ± 3.0	3.6 ± 2.9	3.6 ± 3.1	0.986

Data are summarized as frequency (percentage), mean ± standard deviation or median [interquartile range].

TIA, transient ischaemic attack; HbA1c, glycated haemoglobin; eGFR, estimated Glomerular filtration rate; ALT, alanine aminotransferase; AST, aspartate aminotransferase; hs-CRP, high sensitivity C-reactive protein; BSA, body surface area; E/A, E-wave velocity/A-wave velocity;

**Table 2:** Performance of RSF models with different numbers of predictors according to the rankings of the initial RSF model in the training cohort (*n* = 1450)

Features	VIMP (%)	Rank of VIMP	Minimum depth	Number of features	AUC, % (95% CI)
1. Age	5.69	1	2.60	Top 1	63.8 (60.7–66.9)
2. NT-proBNP	4.61	2	2.71	Top 2	80.8 (78.8–82.8)
3. Albumin	2.40	3	3.95	Top 3	82.5 (80.6–84.4)
4. Fasting glucose	2.35	4	4.41	Top 4	84.6 (82.8–86.3)
5. eGFR	2.19	5	2.94	Top 5	85.3 (83.6–87.0)
6. Uric acid	2.19	6	3.95	Top 6	86.6 (85.0–88.1)
7. Left atrial size	1.40	7	3.89	Top 7	87.6 (86.1–89.1)
8. Peripheral artery disease	1.38	8	8.80	Top 8	87.7 (86.2–89.2)
9. Left ventricular ejection fraction	1.26	9	4.23	Top 9	88.2 (86.7–89.7)
10. hsCRP	0.97	10	5.07	Top 10	88.9 (87.4–90.4)
11. TRPG	0.96	11	4.11	Top 11	89.5 (88.1–90.9)
12. Atrial fibrillation	0.96	12	6.54	Top 12	89.5 (88.1–90.9)
13. Systolic blood pressure	0.96	13	4.94	Top 13	90.1 (88.7–91.5)
14. LVMI	0.89	14	5.69	Top 14	90.3 (89.0–91.7)
15. AST/ALT	0.87	15	4.62	Top 15	90.6 (89.3–91.9)
16. LVEDD	0.69	16	4.84	Top 16	90.9 (89.6–92.1)
17. High-density lipoprotein	0.61	17	4.91	Top 17	91.3 (90.1–92.5)
18. Low-density lipoprotein	0.59	18	5.16	Top 18	91.6 (90.4–92.8)
19. Body mass index	0.57	19	5.40	Top 19	91.8 (90.7–92.9)
20. HbA1C	0.57	20	4.53	Top 20	91.9(90.8–93.1)
21. Myocardial infarction	0.56	21	10.03	Top 21	91.9 (90.8–93.1)
22. Chronic obstructive pulmonary disease	0.43	22	11.76	Top 22	92.1 (91.0–93.2)
23. Male	0.12	23	11.08	Top 23	91.8 (90.6–92.9)
24. Stroke	−0.01	24	10.37	Top 24	91.9 (90.8–93.1)
25. LVH	−0.04	25	12.66	Top 25	92.0 (90.9–93.1)
26. Coronary artery disease	−0.12	26	10.65	Top 26	92.0 (90.9–93.1)
27. Previous coronary artery bypass graft	−0.15	27	10.22	Top 27	92.2 (91.0–93.3)

RSF, random survival forest; VIMP, variable importance; AUC, area under curve; CI, confidence interval; NT-proBNP, N-terminal prohormone of brain natriuretic peptide; eGFR, estimated glomerular filtration rate; TRPG, tricuspid regurgitation peak gradient; LVMI. left ventricular mass index; AST, aspartate aminotransferase; ALT, alanine aminotransferase; LVEDD, left ventricular end-diastolic diameter; HbA1c, glycated haemoglobinx`; LVH, left ventricular hypertrophy.

### Statistical analysis

Baseline characteristics were compared between outcome and non-outcome groups using chi-square tests for categorical variables and *t*-tests or Mann–Whitney *U* tests for continuous variables. Missing data were imputed using the Expectation-Maximization algorithm. Model performance was assessed using AUCs for both the initial 27-variable and final 9-variable RSF models, with external validation conducted in an independent cohort. Patients were stratified into four groups (0–1, 2–3, 4–5, ≥6 risk factors) based on the selected predictors; log-rank tests for trend and unadjusted Cox models (reference: 0–1 feature) were used to examine prognostic impact. RSF models were implemented in R (v4.3.2, randomForestSRC package), and other analyses used SAS (v9.4). A two-sided *P* < 0.05 was considered significant.

## Results

### Baseline characteristics of diabetic HFpEF patient with and without events and outcomes

A total of 1450 patients diagnosed with both HFpEF and DM in the training cohort were included in the analysis. *Table 1* presents the baseline characteristics of diabetic patients with HFpEF, stratified by the occurrence of the composite outcome of HHF or CV death. In the training cohort of diabetic patients with HFpEF, 327 individuals (22.6%) experienced the composite outcome of HHF or CV death during a mean follow-up of 3.6 ± 3.0 years, resulting in an incidence of 6.5 events per 100 person-years (95% confidence interval [CI]: 5.8–7.2). Among these, 218 patients (15.0%) died from CV causes and 137 (9.5%) were HHF (see Supplementary material online, *Table S1*). Compared to those without events, patients in the event group were older (74.9 vs. 73.1 years, *P* = 0.013) and had higher rates of hypertension, coronary artery disease (CAD), peripheral artery disease (PAD), CKD, and atrial fibrillation, but less chronic obstructive pulmonary disease. They also exhibited higher N-terminal pro-brain natriuretic peptide (NT-proBNP) (3266 vs. 2279 pg/mL, *P* = 0.002), uric acid (7.5 vs. 6.8 mg/dL, *P* < 0.001), and larger left atrial size (4.3 vs. 4.1 cm, *P* = 0.001), with no significant differences in other echocardiographic parameters.

As shown in Supplementary material online, *Table S2*, baseline use of most glucose-lowering therapies was similar between groups, except for lower sodium-glucose co-transporter 2 (SGLT2) inhibitor use in the event group (1.2% vs. 3.5%, *P* = 0.036). CV medications more frequently prescribed in the event group included clopidogrel, ACE inhibitors/ARBs, digoxin, and sacubitril/valsartan.

### RSF model and selection of outcome predictors

**Figure 1:**
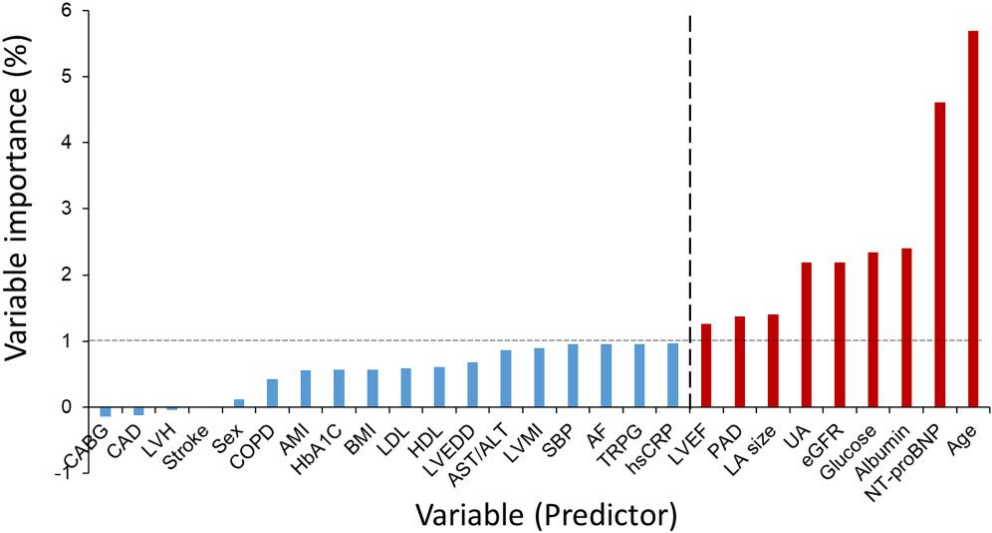
Variable importance ranking. Variable importance scores of the initial 27 candidate predictors in the RSF model. The top 9 variables with the highest importance were retained for the final model construction.

**Figure 2:**
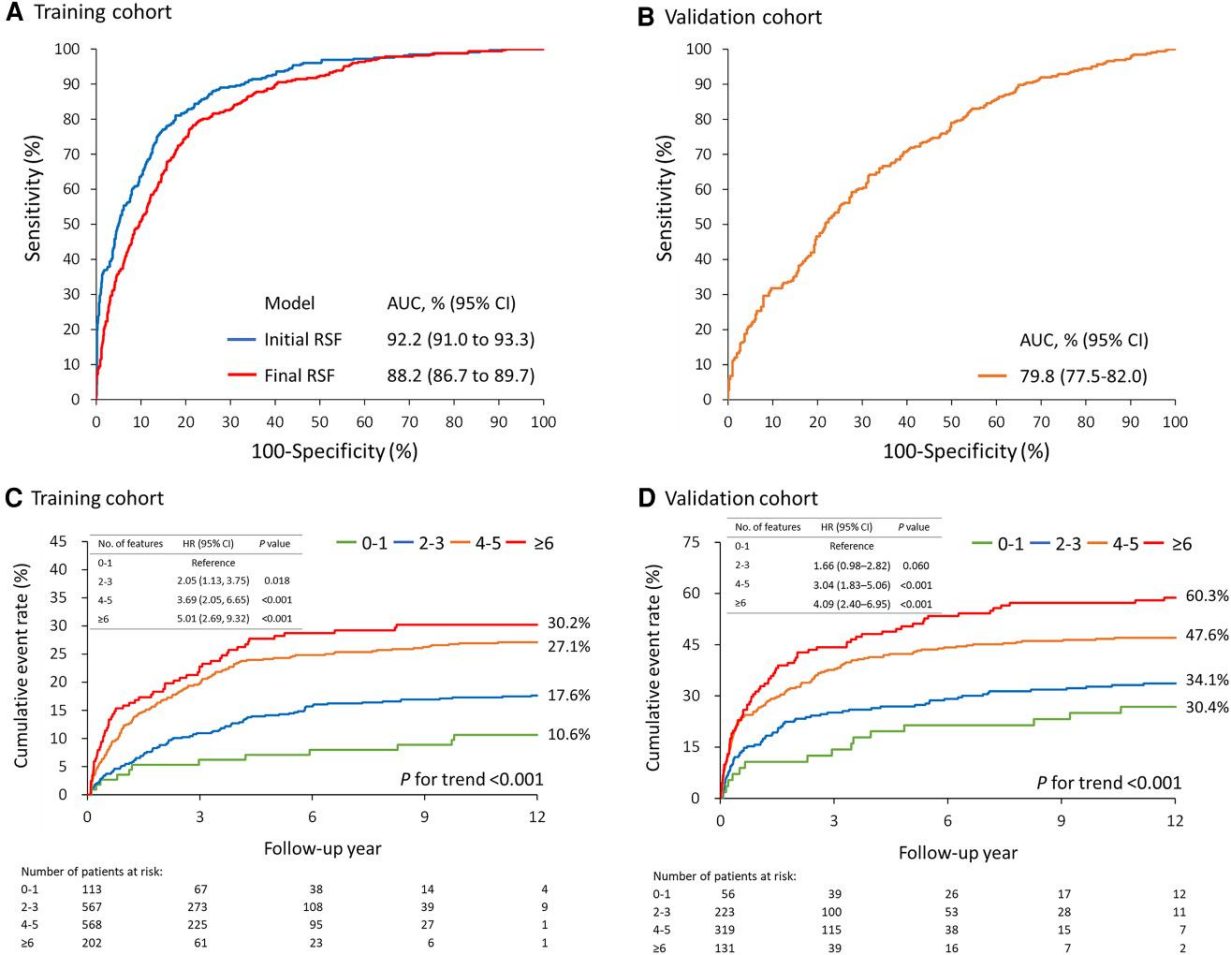
Discriminatory performance and prognostic implications of the RSF model. (*A*) Performance comparison of the initial RSF model (27 variables) and the final RSF model (9 selected variables) in the training cohort. (*B*) Area under the curve (AUC) of the final RSF model in the validation cohort. (*C–D*) Cumulative incidence of the composite outcome of heart failure hospitalization and cardiovascular death, stratified by the number of risk factors (from the 9 selected predictors) in the training (*C*) and validation (*D*) cohorts. Risk thresholds were derived from partial dependence plots using maximal log-rank statistics.

The initial RSF model was established using all 27 variables from the data of the training cohort (*Figure 1*). The VIMPs of the 27 candidate predictors are summarized in *Table 2*. Among them, age emerged as the most influential variable in predicting the composite outcome of HHF or CV death, with a VIMP of 5.7%. This was followed by NT-proBNP (4.6%), serum albumin (2.4%), fasting glucose (2.4%), eGFR (2.2%), uric acid (2.2%), left atrial size (1.4%), PAD (1.4%), and LVEF (1.3%). These top nine variables were selected for inclusion in the final RSF model based on their strong prognostic value and contribution to model performance, achieving an AUC of 88.2% (95% CI: 86.7–89.7%) in the training cohort. *Figure 2A* demonstrates the strong discriminatory performance of the initial RSF model with 27 variables, which achieved an AUC of 92.2% (95% CI: 91.0–93.3%). *Table 2* presents the AUCs for all RSF models constructed using an incremental number of top-ranked predictors from the initial model. In the external validation cohort, the final RSF model incorporating the top 9 variables maintained robust performance, with an AUC of 79.8%, supporting its generalizability (*Figure 2B*). Partial dependence plots for these nine predictors are shown in Supplementary material online, *Figure S2*, highlighting the nonlinear associations between each variable and the risk of the composite outcome after accounting for interactions among predictors.

To enhance clinical applicability, we derived a mnemonic-based risk score, **GUARDIAN-P**, which encapsulates the nine most predictive variables associated with an increased risk of heart failure hospitalization or cardiovascular death, as identified by the RSF model: **G**lucose (fasting ≥166 mg/dL), **U**ric acid (≥7 mg/dL), **A**ge (≥65 years), **R**enal function (eGFR <60 mL/min/1.73 m^2^), **D**ilatation of left atrium (≥4.0 cm), **I**mpaired contractility (LVEF <60%), **A**lbumin <3.5 g/dL, **N**T-proBNP (≥5000 pg/mL) and **P**AD. The acronym simplifies recall and facilitates bedside risk assessment in patients with HFpEF and DM. Each component of the GUARDIAN-P score was determined based on clinically meaningful cut-offs derived from partial dependence plots and validated in survival analyses. These cut-offs were further supported by Kaplan–Meier survival analyses in the training cohort (see Supplementary material online, *Figures S2A*–*I*).

### Prognostic implications of risk factors

A stepwise increase in the cumulative incidence of HHF or CV death was observed with a greater number of risk predictors in both cohorts (*Figure 2C* and *D*). In the training cohort, event rates were 10.6%, 17.6%, 27.1%, and 30.2% among patients with 0–1, 2–3, 4–5, and ≥6 risk factors, respectively, showing a significant trend (log-rank *P* < 0.001; *Figure 2C*). Similarly, in the validation cohort, the corresponding event rates were 30.4%, 34.1%, 47.6%, and 60.3%, respectively, also demonstrating a strong graded association between the number of risk factors and clinical outcomes (log-rank *P* < 0.001; *Figure 2D*). These findings confirm the robust calibration and predictive utility of the final RSF model across different risk strata.

Supplementary material online, *Table S3* compares the distribution of the nine final predictors and follow-up duration between the training cohort (National Taiwan University Hospital) and the validation cohort (MacKay Memorial Hospital). Compared to the training cohort, patients in the validation cohort were slightly younger (72.2 ± 12.7 vs. 73.5 ± 11.8 years, *P* = 0.016) and had a significantly higher prevalence of PAD (16.7% vs. 10.7%, *P* < 0.001). They also exhibited markedly higher fasting glucose levels (180.7 ± 86.1 vs. 135.5 ± 48.5 mg/dL, *P* < 0.001), higher NT-proBNP concentrations (median [IQR]: 4610 [1320–12 200] vs. 2885 [944–6549] pg/mL, *P* < 0.001), and lower eGFR (45.1 ± 28.9 vs. 52.8 ± 31.4 mL/min/1.73 m^2^, *P* < 0.001). In addition, they had smaller left atrial size (3.6 ± 0.5 vs. 4.1 ± 0.6 cm, *P* < 0.001) and lower LVEF (64.1 ± 6.4% vs. 65.3 ± 8.4%, *P* < 0.001). Serum albumin and uric acid levels were comparable between groups. Notably, the validation cohort had a significantly longer mean follow-up duration compared to the training cohort (6.2 ± 4.8 vs. 3.6 ± 3.0 years, *P* < 0.001), which may partially account for the higher cumulative event rate observed in this group. In the validation sample, 324 individuals (44.4%) experienced the composite outcome and the incidence rate of outcome in the validation sample was 13.4 events per 100 person-years (95% CI: 12.0–14.9).

## Discussion

HFpEF is a complex syndrome with various phenotypes, one of which is characterized by the presence of DM.^15^ In Asian populations in particular, HFpEF often exhibits a cardiometabolic phenotype with a high burden of metabolic comorbidities such as DM despite relatively lower levels of obesity compared with Western cohorts.^3^ DM contributes to HFpEF through a multifaceted pathophysiology involving structural, inflammatory, and metabolic derangements.^16^ Key mechanisms include adverse left ventricular remodelling with increased myocardial stiffness and fibrosis, chronic inflammation with endothelial dysfunction, and metabolic abnormalities in the diabetic myocardium.^17^ In this study, we developed and externally validated a ML-based risk prediction model for HHF and CV death in patients with DM and HFpEF. Using an RSF approach applied to electronic health record data, we identified nine key clinical variables that provided robust predictive performance across both training and validation cohorts. The final model achieved high discrimination with an AUC of 88.2% in the training cohort and 79.8% in the validation cohort, highlighting its potential clinical utility for risk stratification in this vulnerable population.

Multiple studies have investigated the distinct clinical profile of HFpEF in patients with DM and its impact on outcomes. In the CHARM (Candesartan in Heart Failure: Assessment of Reduction in Mortality and Morbidity) programme, participants with LVEF above 40% and DM had a two-fold higher risk of CV death or HHF, along with an approximately 80% elevated risk of all-cause mortality after adjustment for confounders.^18^ Similarly, the DIG (Digitalis Investigation Group) trial, which included patients with LVEF greater than 45%, reported a 68% increase in the adjusted risk of heart failure-related death or hospitalization in those with DM.^19^ The RELAX trial (Phosphodiesterase-5 Inhibition to Improve Clinical Status and Exercise Capacity in HFpEF), though smaller in scale and limited to patients with LVEF ≥50%, employed a comprehensive phenotyping strategy using biomarkers, echocardiography, cardiac MRI, and exercise testing to further elucidate the diabetic HFpEF phenotype. HFpEF patients with DM have higher hospitalization risk and reduced exercise capacity, likely due to multimorbidity, left ventricular hypertrophy, chronotropic incompetence, and activation of inflammatory and profibrotic pathways.^20^ In the I-PRESERVE trial (LVEF ≥45%), patients with DM showed higher rates of all-cause mortality and HHF, along with more severe cardiac remodelling despite similar LVEF. These adverse outcomes persisted after adjusting for clinical factors, and were more pronounced in those receiving insulin therapy.^21^ The severity and duration of DM further influence outcomes in HFpEF. A TOPCAT sub-analysis found that patients requiring insulin had nearly 50% higher all-cause and CV mortality than those on oral agents or without DM.^22^ Those with milder, non-insulin-treated DM had outcomes similar to non-DM, suggesting that advanced or poorly controlled DM significantly worsens HFpEF prognosis. Therefore, implementing a risk stratification model in clinical practice is essential to accurately identify high-risk individuals and support clinicians in assessing the likelihood of HHF and CV death in patients with HFpEF and DM.

Several clinical scores have been developed to assess risk in patients with HFpEF or DM, each with distinct purposes and limitations. The HFA-PEFF score, established by the European Society of Cardiology, is a structured diagnostic tool for HFpEF, integrating functional, morphological, and biomarker domains.^23^ Similarly, the H2FPEF score, developed by the Mayo Clinic, is a diagnostic tool that assigns points for six clinical and echocardiographic variables: Heavy (BMI >30), Hypertensive (≥2 antihypertensives), Atrial Fibrillation, Pulmonary hypertension, Elder (age >60), and Filling pressure (E/e’ > 9).^24^ The high BMI component of the H2FPEF score may indirectly reflect several pathophysiological effects associated with DM. However, the score is primarily designed as a diagnostic rather than a prognostic tool. In contrast, the WATCH-DM score and the TRS-HFDM score focus on predicting HF risk in patients with DM. The WATCH-DM score uses common clinical variables (e.g. age, hypertension, creatinine, lipid profiles, fasting glucose, ECG findings, myocardial infarction and CABG),^25^ while TRS-HF_DM_ incorporates prior HF, atrial fibrillation, coronary disease, renal function, and urine albumin/creatinine ratio to stratify HF risk, particularly in diabetic populations.^26^ Collectively, these scores offer valuable frameworks for risk assessment, yet none are tailored specifically for patients with established HFpEF and DM. This emphasizes the necessity for specialized, ML-driven models to improve prognostic accuracy and enable personalized management strategies for this vulnerable population.

Although the WATCH-DM score was also developed using a ML approach with RSF, it was designed to predict incident HF in patients with type 2 DM without pre-existing HF, thus focusing on a prevention-oriented population.^25^ While some studies have applied the WATCH-DM score in HFpEF populations with DM to estimate prognosis, its discriminatory performance was modest, with reported AUCs of 0.64 for 1-year mortality^27^ and 0.71 for CV death.^28^ In contrast, GUARDIAN-P risk score was specifically developed for patients with established HFpEF and DM and achieved higher predictive accuracy, with AUCs of 88.2% in the training cohort and 79.8% in the validation cohort. By incorporating echocardiographic indices, NT-proBNP, and metabolic and renal biomarkers, our RSF-based model offers refined risk stratification tailored to this high-risk group. Unlike the WATCH-DM score, which targets incident HF, our model predicts the composite outcome of HHF and CV death, providing a more clinically actionable tool for guiding care in patients with diabetic HFpEF.

Our findings underscore the prognostic significance of several established and emerging risk markers in diabetic HFpEF patients. Among them, age and NT-proBNP levels were the most influential predictors, aligning with previous studies that linked older age and elevated NT-proBNP to adverse HFpEF outcomes.^10,29^ Markers of metabolic and renal dysfunction—such as elevated fasting glucose, reduced eGFR, and hypoalbuminemia—also emerged as strong contributors, reflecting the complex cardiorenal-metabolic interplay that typifies diabetic HFpEF.^30^ Although HbA1c is a standard marker for assessing long-term glycaemic control in DM, it was not included in the final WATCH-DM score, primarily due to missing data in the external validation cohort.^25^ In contrast, our dataset had complete HbA1c information, yet fasting glucose consistently demonstrated greater prognostic relevance, as reflected by a higher VIMP ranking in the RSF model. This may reflect fasting glucose’s greater sensitivity to acute metabolic stress, which more directly contributes to decompensation and adverse outcomes in HFpEF with DM. In contrast, HbA1c averages glucose over months and can be affected by conditions like anaemia or CKD, reducing its reliability. Additionally, RSF algorithms prioritize variables that create the most informative survival splits, and fasting glucose may better differentiate short-term risk.

Structural cardiac markers also played an important role in risk stratification. Left atrial enlargement and reduced LVEF within the ‘preserved’ range have been associated with increased filling pressures and worse prognosis, consistent with previous findings.^31^ Importantly, these structural parameters likely reflect the cumulative consequences of long-standing cardiometabolic stress, including DM-related myocardial remodelling, rather than representing isolated baseline abnormalities alone. Notably, PAD was a significant risk factor, suggesting that systemic atherosclerotic burden may exacerbate HFpEF pathophysiology in diabetic patients. Although CAD is an established risk factor in HFpEF populations, its variable importance ranking in the RSF model was relatively modest. This may reflect overlapping prognostic information with other markers of CV and systemic disease burden, including NT-proBNP, PAD, renal dysfunction, and LVEF, thereby limiting its additional incremental predictive value in the multivariable model. Uric acid, while traditionally viewed as a bystander, has been increasingly recognized as a marker of oxidative stress and vascular inflammation, and its inverse association with outcome in our model supports its prognostic value.^32^ Compared to traditional Cox models, the RSF approach handles high-dimensional data, missing values, and nonlinear interactions more effectively, making it ideal for real-world clinical use. Partial dependence plots enabled visualization and clinically meaningful dichotomization of continuous variables to enhance practical utility. External validation in an independent cohort from another centre confirmed the model’s generalizability, despite baseline differences. The higher event rates observed in the validation cohort likely reflect a combination of longer follow-up duration, greater baseline disease severity (including higher NT-proBNP levels and worse renal function), and potential differences in referral patterns between tertiary medical centres.

Our study has several limitations. First, the study is retrospective and observational, and although rigorous methods and external validation were applied, causal inference cannot be established. DM duration could not be reliably determined because the initial diagnosis may have occurred outside the healthcare system or prior to database coverage. Medication data were captured at baseline and were not included as predictors because treatment use may vary over time and adherence could not be confirmed longitudinally. Instead, metabolic markers such as fasting glucose and HbA1c were incorporated to reflect disease severity and treatment exposure. However, the model can aid clinicians in identifying high-risk diabetic HFpEF patients who may benefit from more intensive monitoring or early initiation of evidence-based therapies such as SGLT2 inhibitors^33^ and GLP-1 RAs.^34^ Second, external validation was limited to a single medical centre in Taiwan, and further testing in other healthcare systems and populations is needed.

## Conclusions

We developed and externally validated an ML–based risk prediction model using an RSF approach to estimate the risk of HHF and CV death in patients with DM and HFpEF. The final model, comprising nine readily available clinical variables (GUARDIAN-P), demonstrated strong discriminatory performance and generalizability across independent hospital systems.

## Supplementary Material

ztag083_Supplementary_Data

## Data Availability

The datasets analysed in this study were derived from the National Taiwan University Hospital–Integrated Medical Database (NTUH-iMD). These data contain sensitive patient information and are therefore not publicly available. However, de-identified data that support the findings of this study are available from the corresponding author upon reasonable request and with approval from the National Taiwan University Hospital Research Ethics Committee.
