# Supplementary material for: Machine learning–based prediction of mortality and hospitalization in diabetic patients with heart failure with preserved ejection fraction: the GUARDIAN-P risk score
Source: Eur Heart J Digit Health. 2026 Jun 10;7(6):ztag083. doi: 10.1093/ehjdh/ztag083 (PMC13293260; doi:10.1093/ehjdh/ztag083)
Supplement: ztag083_Supplementary_Data [file ztag083_supplementary_data.zip › Supplementary materials.docx]

**Supplementary materials**

**Validation Cohort**

The validation heart failure with preserved ejection fraction (HFpEF) cohort was derived from two tertiary centers (Taipei and Tamsui branches of MacKay Memorial Hospital) as part of the Echocardiography Core Laboratory database, spanning January 2008 to May 31, 2018. Consecutive patients with HFpEF [left ventricular ejection fraction (LVEF) ≥ 50% and no prior history of LVEF < 50%] presenting with worsening heart failure or hospitalization for heart failure (HHF) were enrolled if they met natriuretic peptide criteria at the time of the event: B-type natriuretic peptide (BNP) ≥ 100 pg/mL or N-terminal pro-BNP (NT-proBNP) ≥ 300 pg/mL in sinus rhythm, or BNP ≥ 200 pg/mL or NT-proBNP ≥ 900 pg/mL in atrial fibrillation. Diabetes mellitus (DM) status was determined through electronic chart review based on the latest American Diabetes Association criteria^1^, including the presence or absence of glucose-lowering therapies.

**Predictive behaviour of the RSF variables**

The association between the risk of the composite outcome, heart failure hospitalization and cardiovascular death, and each of the nine selected predictors was explored using partial dependence plots. These plots were generated through the following steps: (1) fitting the random survival forest (RSF) model with the nine selected features; (2) selecting one variable of interest while holding the other eight constants; (3) computing the predicted outcome across a range of values for the selected variable; and (4) plotting these values on the x-axis against the corresponding average predicted risks on the y-axis to visualize the relationship^2,3^. Partial dependence plots are particularly useful for capturing and illustrating nonlinear effects of individual predictors on the outcome^4^. Based on the patterns observed in these plots and clinical relevance, each of the nine variables was dichotomized for further analysis. For example, in the case of age, a noticeable decline in predicted survival was observed beginning around 60–65 years (**Supplemental Figure 2A**). Given the conventional threshold defining elderly individuals at 65 years, age was dichotomized at this cutoff. Kaplan–Meier survival curves and log-rank tests were then used to assess the prognostic significance of these dichotomized subgroups for each variable (**Supplemental Figures 2A to 2I**).

**Reference:**

1. Elsayed NA, Aleppo G, Bannuru RR*, et al.* 2. Diagnosis and Classification of Diabetes:<i>Standards of Care in Diabetes—2024</i>. *Diabetes Care* 2024;**47**:S20-S42. doi: 10.2337/dc24-s002

2. Friedman JH. Greedy function approximation: A gradient boosting machine. *The Annals of Statistics* 2001;**29**:1189-1232. doi: 10.1214/aos/1013203451

3. Goldstein A, Kapelner A, Bleich J, Pitkin E. Peeking Inside the Black Box: Visualizing Statistical Learning With Plots of Individual Conditional Expectation. *Journal of Computational and Graphical Statistics* 2015;**24**:44-65. doi: 10.1080/10618600.2014.907095

4. Dietrich S, Floegel A, Troll M*, et al.* Random Survival Forest in practice: a method for modelling complex metabolomics data in time to event analysis. *Int J Epidemiol* 2016;**45**:1406-1420. doi: 10.1093/ije/dyw145

# Supplemental Table

**Supplemental** **Table S1**. Clinical outcomes in patients with diabetes in the training cohort

|  | Total  (*n* = 1,450) | |
| --- | --- | --- |
| Outcome | Event rate (%) | Incidence (95% CI) † |
| Hospitalization for heart failure | 137 (9.5) | 2.7 (2.3, 3.2) |
| Cardiovascular death | 218 (15.0) | 4.0 (3.5, 4.5) |
| Heart failure or cardiovascular death | 327 (22.6) | 6.5 (5.8, 7.2) |
| All-cause mortality | 780 (53.8) | 14.4 (13.4, 15.4) |

Abbreviation:

† Number of events per 100 person-years.

**Supplemental** **Table S2**. Baseline medications in patients with diabetes and HFpEF who suffered from heart failure outcome (the composite of heart failure hospitalization and cardiovascular death) and who did not

| Variable | Available number | Total  (*n* = 1,450) | Event  (*n* = 327) | Event-free  (*n* = 1,123) | *P* value |
| --- | --- | --- | --- | --- | --- |
| Baseline medications |  |  |  |  |  |
| Sulfonylureas | 1,450 | 531 (36.6) | 130 (39.8) | 401 (35.7) | 0.181 |
| Thiazolidinediones | 1,450 | 112 (7.7) | 28 (8.6) | 84 (7.5) | 0.519 |
| Metformin | 1,450 | 536 (37.0) | 126 (38.5) | 410 (36.5) | 0.505 |
| Meglitinides | 1,450 | 113 (7.8) | 28 (8.6) | 85 (7.6) | 0.555 |
| DPP4i | 1,450 | 487 (33.6) | 101 (30.9) | 386 (34.4) | 0.240 |
| SGLT2i | 1,450 | 43 (3.0) | 4 (1.2) | 39 (3.5) | **0.035** |
| GLP1 | 1,450 | 11 (0.8) | 0 (0.0) | 11 (1.0) | 0.072 |
| Insulin | 1,450 | 721 (49.7) | 157 (48.0) | 564 (50.2) | 0.482 |
| Statin | 1,450 | 618 (42.6) | 128 (39.1) | 490 (43.6) | 0.149 |
| Fibrate | 1,450 | 63 (4.3) | 13 (4.0) | 50 (4.5) | 0.710 |
| Aspirin | 1,450 | 606 (41.8) | 148 (45.3) | 458 (40.8) | 0.149 |
| Clopidogrel | 1,450 | 319 (22.0) | 85 (26.0) | 234 (20.8) | **0.048** |
| Prasugrel | 1,450 | 1 (0.1) | 0 (0.0) | 1 (0.1) | 0.589 |
| Ticagrelor | 1,450 | 27 (1.9) | 7 (2.1) | 20 (1.8) | 0.672 |
| Warfarin | 1,450 | 54 (3.7) | 14 (4.3) | 40 (3.6) | 0.545 |
| Novel oral anticoagulants | 1,450 | 120 (8.3) | 21 (6.4) | 99 (8.8) | 0.167 |
| Alpha blocker | 1,450 | 296 (20.4) | 65 (19.9) | 231 (20.6) | 0.785 |
| Beta blocker | 1,450 | 801 (55.2) | 176 (53.8) | 625 (55.7) | 0.558 |
| ACEi/ARB | 1,450 | 843 (58.1) | 208 (63.6) | 635 (56.5) | **0.023** |
| CCB | 1,450 | 867 (59.8) | 194 (59.3) | 673 (59.9) | 0.845 |
| Direct vasodilator (hydralazine) | 1,450 | 140 (9.7) | 35 (10.7) | 105 (9.3) | 0.466 |
| Nitrate | 1,450 | 582 (40.1) | 128 (39.1) | 454 (40.4) | 0.677 |
| Loop diuretic | 1,450 | 929 (64.1) | 208 (63.6) | 721 (64.2) | 0.844 |
| Thiazide | 1,450 | 290 (20.0) | 72 (22.0) | 218 (19.4) | 0.300 |
| K sparing diuretic | 1,450 | 330 (22.8) | 74 (22.6) | 256 (22.8) | 0.950 |
| Digoxin | 1,450 | 165 (11.4) | 48 (14.7) | 117 (10.4) | **0.033** |
| Sacubitril/valsartan | 1,450 | 13 (0.9) | 6 (1.8) | 7 (0.6) | **0.041** |
| Ivabradine | 1,450 | 10 (0.7) | 1 (0.3) | 9 (0.8) | 0.341 |

Abbreviation: DDP4i, dipeptidyl peptidase-4 inhibitor; SGLT2i, sodium glucose co-transporters 2 inhibitor; GLP1, glucagon-like peptide 1; ACEi, angiotensin-converting enzyme inhibitor; ARB, angiotensin II receptor blocker; CCB, calcium channel blocker; K, potassium;

Data were summarized as frequency (percentage).

**Supplemental Table S3**. The distribution of the nine finally-selected predictors and mean follow up duration between the training (National Taiwan University Hospital) and validation (MacKay Memorial Hospital) cohorts

| Variable | Training cohort  (*n* = 1,450) | Validation cohort  (*n* = 729) | *P* |
| --- | --- | --- | --- |
| Age, year | 73.5 ± 11.8 | 72.2 ± 12.7 | **0.016** |
| Peripheral artery disease | 155 (10.7) | 122 (16.7) | **<0.001** |
| Fasting glucose, mg/dL | 135.5 ± 48.5 | 180.7 ± 86.1 | **<0.001** |
| eGFR, mL/min/1.73m^2^ | 52.8 ± 31.4 | 45.1 ± 28.9 | **<0.001** |
| Albumin, g/dL | 3.59 ± 0.58 | 3.60 ± 0.57 | 0.745 |
| Uric acid, mg/dL | 6.8 ± 2.0 | 7.0 ± 2.1 | 0.088 |
| NT-proBNP, pg/mL | 2885 [944, 6549] | 4610 [1320, 12200] | **<0.001** |
| Left atrial size, cm | 4.1 ± 0.6 | 3.6 ± 0.5 | **<0.001** |
| LVEF, % | 65.3 ± 8.4 | 64.1 ± 6.4 | **<0.001** |
| Follow-up year | 3.6 ± 3.0 | 6.2 ± 4.8 | **<0.001** |

Abbreviation: eGFR, estimated Glomerular filtration rate; NT-proBNP, N-terminal prohormone of brain natriuretic peptide; LVEF, left ventricular ejection fraction;

Data were summarized as mean ± standard deviation or frequency (percentage).





**Supplemental Figure 1. Study Flowchart.**

Flow diagram outlining cohort selection and analysis steps.

Abbreviations: DM, diabetes mellitus; HF, heart failure; HFpEF, heart failure with preserved ejection fraction; HHF, hospitalization for heart failure; LVEF, left ventricular ejection fraction.


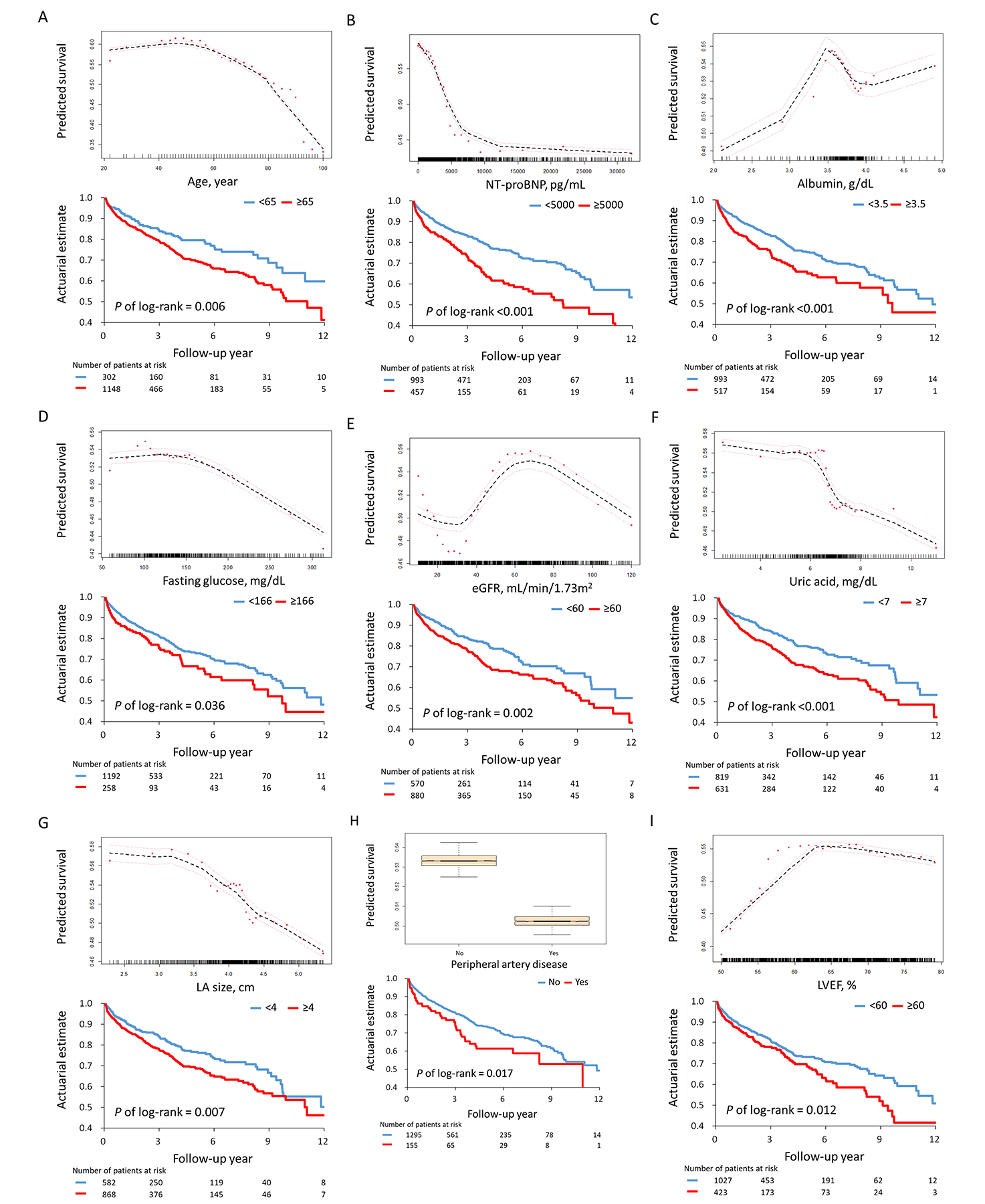


**Supplemental Figure 2. Partial Dependence Plots of Predictors.**

Partial dependence plots illustrating the marginal effect of each of the 9 selected predictors from the final random survival forest model on the risk of the composite outcome in the training cohort.

Abbreviations: NT-proBNP, N-terminal pro–B-type natriuretic peptide; eGFR, estimated glomerular filtration rate; LA, left atrium; LVEF, left ventricular ejection fraction; PAD, peripheral artery disease.
