# Supplementary figures and images for: Machine learning–based prediction of mortality and hospitalization in diabetic patients with heart failure with preserved ejection fraction: the GUARDIAN-P risk score
Source: Eur Heart J Digit Health. 2026 Jun 10;7(6):ztag083. doi: 10.1093/ehjdh/ztag083 (PMC13293260; doi:10.1093/ehjdh/ztag083)

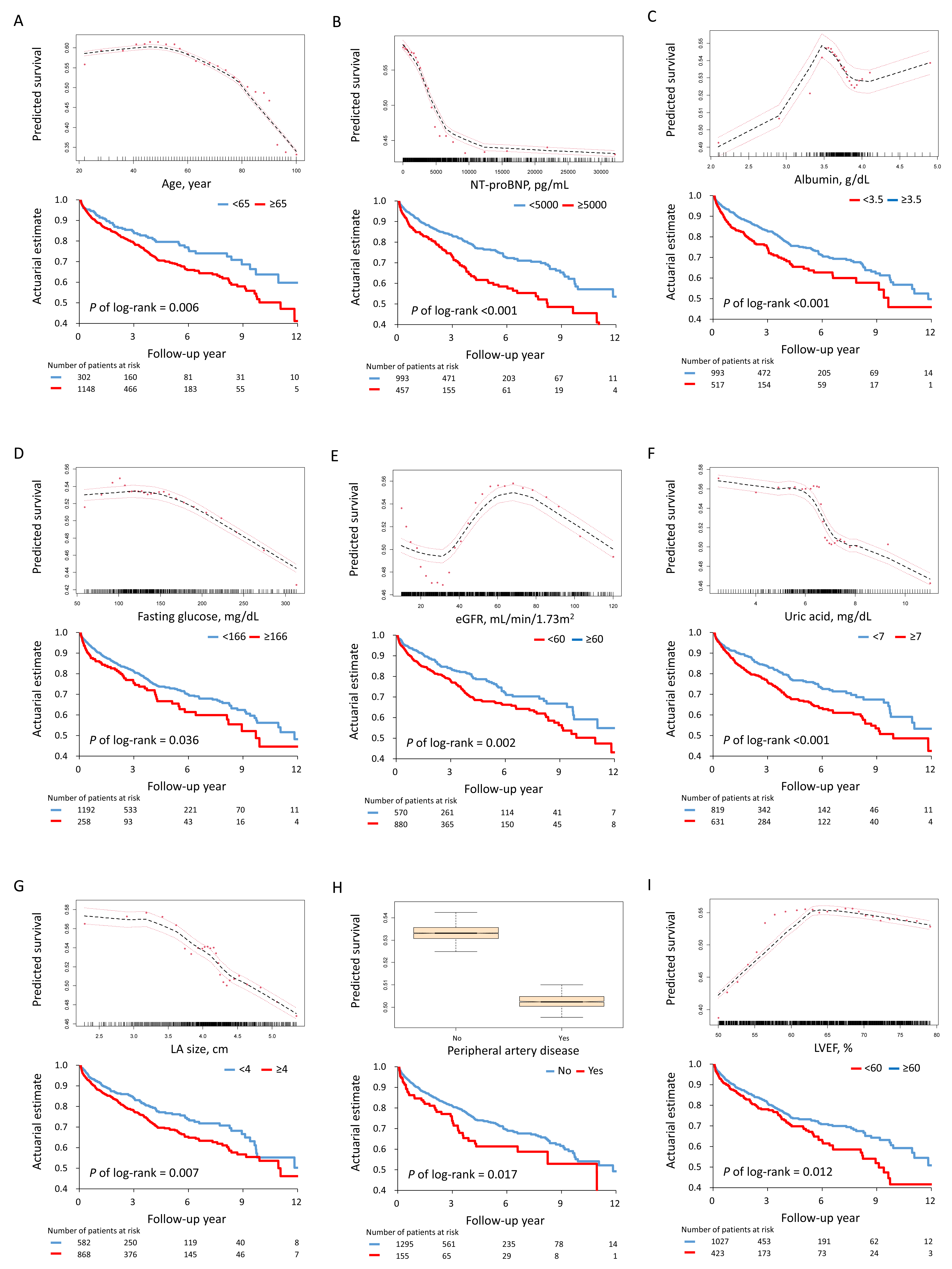

Supplement: ztag083_Supplementary_Data [file ztag083_supplementary_data.zip › Supplementary Figure 2.tif]
